# Promoting Youth Mental Wellbeing: A Photovoice Project with Adolescents and Young Adults in the Hospital Context

**DOI:** 10.3390/ijerph22040648

**Published:** 2025-04-20

**Authors:** Federica Graziano, Federica Toppino, Lisa Vennettillo, Giovanni Abbate Daga, Deborah Concas, Giulia Mazzone, Paola Quarello, Guido Teghille, Giulia Zucchetti, Chiara Davico

**Affiliations:** 1 Department of Psychology, University of Turin, 10124 Turin, Italy; 2 Eating Disorders Center, Department of Neuroscience “Rita Levi Montalcini”, University of Turin, 10126 Turin, Italy; federica.toppino@unito.it (F.T.); lisa.vennettillo@unito.it (L.V.); giovanni.abbatedaga@unito.it (G.A.D.); 3 Pediatric Onco-Hematology Division, Regina Margherita Children’s Hospital, University of Torino, 10126 Turin, Italy; psicologia.concasdeborah@gmail.com (D.C.); paola.quarello@unito.it (P.Q.); giulia.zucchetti@unito.it (G.Z.); 4 Section of Child and Adolescent Neuropsychiatry, Department of Public Health and Pediatric Sciences, University of Turin, 10126 Turin, Italy; mazzone.giulia92@gmail.com (G.M.); guido.teghille@unito.it (G.T.); chiara.davico@unito.it (C.D.)

**Keywords:** adolescents, young adults, wellbeing, mental health, Photovoice, participatory research, adolescent psychiatry, eating disorders, thematic analysis

## Abstract

Given the importance of youth mental health for public policy, it is crucial to involve young people directly in participatory research to investigate their views and translate their demands into concrete actions. The aim of the study was to define the concept of mental wellbeing as perceived by a group of adolescent and young adult patients in two large hospitals in northwestern Italy and to find out, together with them, what institutions can do to promote youth wellbeing. Thirty-nine participants (13–25 years old, 90% female), divided into four groups, took part in a Photovoice workshop. Individual interviews were conducted with 21 participants and the transcripts were thematically analyzed. The photos were categorized into five themes: nature, traveling, passions and leisure, relationships, and animals. The definition of mental wellbeing can be traced back to eight themes: sharing experiences and emotions with others, calm and tranquility, personal fulfilment, sense of belonging, pleasant physical sensations, freedom and discovery, involvement and commitment, and happiness. The key recommendations for promoting mental wellbeing were providing information about mental health, tackling the stigma of mental illness, and providing psychological support in school and health services. The implications of these findings for policy makers involved in planning health services for young people are discussed.

## 1. Introduction

In the period after the COVID-19 pandemic, an increase in behavioral and emotional problems was observed in adolescents and young adults, which was consistent with a trend already observed before the pandemic [1,2]. In particular, an increase in suicidality, eating disorders, anxiety, and depression was observed in the post-pandemic period, especially among females [3,4], worldwide and also in Italy [5,6]. These findings were confirmed by various systematic reviews of studies conducted during and immediately after the COVID-19 pandemic [7,8].

The widespread occurrence of mental health problems among adolescents is a major public health concern as the risk of mental illness, problematic social relationships, and poor psychosocial adjustment has increased among young people. For these reasons, it is important to provide psychological support and care and to actively promote those conditions that can counteract psychological malaise and promote wellbeing. Promoting the emotional, social, and psychological wellbeing of adolescents and young adults should therefore be considered a priority for public health policy. This promotion is part of a broad debate on the need to include more research on protective factors as a way to contain stress and psychiatric symptoms [9,10].

Studies in positive psychology have paid increasing attention to the concept of wellbeing. They have attempted to outline a definition of this multidimensional construct by integrating two perspectives: the hedonic approach, which focuses mainly on wellbeing in terms of pleasure and happiness, and the eudaimonic approach, which defines wellbeing in terms of the individual’s sense of personal fulfilment and self-realization [11].

For adolescents and young adults, the definition of wellbeing cannot be separated from the fulfillment of the developmental tasks typical for these periods of life. The main developmental tasks of adolescents are related to redefining their identity, negotiating independence from the family, and developing a sense of belonging to the peer group [12], whereas young adults mainly face developmental tasks related to occupational and economic independence, intimate partner relationships, and parenthood [13] Therefore, social contexts, especially the family, peer group, and school experiences, play an important role in defining adolescents’ and young adults’ wellbeing. For adolescents and young adults with psychiatric problems (e.g., anxiety disorders, suicidality and self-harm, and eating disorders) or physical illnesses with significant psychological effects (e.g., oncological diseases), the definition of wellbeing is even more complicated. These patients must cope with the developmental tasks of their age under conditions of physical and/or psychological suffering and often experience social isolation during long hospital stays, which has a significant impact on their mental wellbeing.

Given the importance of these aspects, it is crucial to involve young people in the process of defining the concept of wellbeing. From the perspective of participatory research, the direct involvement of adolescents and young adults makes it possible to understand their view of the concept of wellbeing and to explore their proposals for its promotion. In particular, involving young patients in participatory research can provide specific insights into the context of their lives (family, school, peer group, and wider community) but also into the context of care (hospital and health services) that they directly experience [14,15]. In a participatory research approach, adolescents and young adults are not only regarded as objects of study but also as active participants in the research process. Through their direct involvement, they can help shape the content of the research and engage in an active dialogue with institutions and the adult world, which, too often, pays too little attention to their voices [16]. Participatory research approaches make it possible to translate young people’s demands into concrete measures to change and promote mental health in different life contexts. Adolescent and young adult patients, in particular, have relevant experiential knowledge derived from their lived experience in the hospital context [17]. Taking their perspective into account can help develop health policies and promote care contexts that are more responsive to their needs and better support their development [15,16].

Among participatory research approaches, we chose Photovoice for this study, a participatory action research method in which people are invited to take photos on a specific, often sensitive topic and then describe their experiences, usually in group discussions. Photovoice is primarily used in health research to actively engage participants in research and discussion, promote personal empowerment, and drive change in the community [18]. This method has been shown to be particularly suitable for vulnerable groups of participants [19,20] and encourages the sharing of individual experiences on sensitive topics such as mental health [21,22]. In particular, the Photovoice method has been recognized as a useful approach for youth engagement and advocacy in the field of youth mental health [23].

The aims of this study were as follows:

1. Define the concept of mental wellbeing as perceived by a group of adolescents and young adults in a hospital context (child and adolescent neuropsychiatry unit, oncohematology unit, and eating disorders treatment center);
2. Investigate what educational and health care institutions can do to promote the wellbeing of adolescents and young adults according to the participants involved.

Due to the explorative nature of the participatory research approach, no hypotheses were formulated. The Photovoice method was used to encourage active involvement of participants, facilitate group discussions on the sensitive topic of youth mental wellbeing, and develop recommendations for policy makers responsible for youth health policy.

## 2. Materials and Methods

### 2.1. Study Participants

This study was part of a broader Italian interdisciplinary participatory research to design social policies in response to the effects of the COVID-19 pandemic on the wellbeing of children and adolescents. The study included as insightful participants a convenience sample of adolescent and young adult patients in two large university hospitals in an urban area in northwestern Italy. Specifically, adolescent patients were included from two departments of the pediatric hospital: the neuropsychiatry unit, where most patients were admitted for anxiety, suicidality, or eating disorders, and the oncohematology unit. Young adults came instead from the eating disorders treatment center of the adult hospital. Patients from the neuropsychiatric unit are treated as inpatients for an average of thirty days and then spend an average of six months in the day hospital where they receive twice-weekly psychiatric examinations, individual psychotherapy, and group rehabilitation interventions. Patients from the oncohematology unit, after hospitalization, participate in day hospital activities, followed by outpatient check-ups, the duration of which varies and is specific depending on the clinical condition. Patients in both units can continue their school activities via the hospital school. The day hospital activities of the eating disorder center last an average of 6 months and take place 5 days a week and include psychiatric assessments, individual and group psychotherapy, group rehabilitation activities, supervised meals and nutritional counselling.

This purposive sampling allowed us to reach young people who were in a particularly vulnerable physical and psychological condition and had direct experience of health and care services.

First, the researchers introduced the study to the medical staff at each facility and involved them in recruiting study participants. Inclusion criteria for participants were that they were between the ages of 13 and 25 and were interested in participating in the Photovoice workshop and in group activities for the duration of the study. A further criterion for oncohematology cancer outpatients was being in the off-therapy phase, i.e., having overcome the most critical phase of the disease.

The participants signed an informed written consent to participate in the study. For underage patients (under 18 years of age), written parental consent was obtained. The study was approved by the Hospital Ethics Committee, protocol number 0148744, date of approval 19 December 2023.

The study included 39 participants (mean age = 17.9, sd = 4; 90% female) divided into 4 groups (2 groups from the department of child and adolescent neuropsychiatry and from the day hospital, respectively; 1 group from the department of oncohematology and 1 group from the treatment center for eating disorders). The characteristics of the participants are summarized in Table 1.

### 2.2. Procedure: The Photovoice Workshop

The Photovoice workshop was entitled “Say it with a photo!”. Each of the four groups participated in five meetings, which took place from January to April 2024.

a. First and second meetings. The first preparatory meeting was used to introduce the study, get to know the participants, and discuss young people’s wellbeing, based on reading newspapers and research articles about young people’s health. The second meeting focused on the use of photos to convey thoughts and the symbolic value of images. A discussion was held based on example images brought by researchers. Then participants were asked to take 2–4 photos that represented their own idea of mental wellbeing. No further instructions were given except that no individuals were allowed to be photographed to protect their privacy.
b. Taking photos. The photos were taken over a two-week period and then emailed to the researchers. The researchers printed out the photos of all groups and brought them to subsequent meetings. Since the patients of the adolescent psychiatric ward were not allowed to use smartphones or cameras according to hospital regulations, we adapted flexibly to these circumstances and decided with participants to create collages that depicted their ideas of mental wellbeing. The researchers photographed the adolescents’ collages, and they were allowed to keep their collages at the end of the workshop.
c. Individual interviews. Prior to the third group meeting of the workshop, all participants were asked if they agreed to be interviewed individually from the photo taken. Individual interviews are often conducted in Photovoice workshops and combined with group discussions, especially when vulnerable people are invited to talk about their personal experiences [24,25,26]. Twenty-one participants agreed to be interviewed (seven from child and adolescent neuropsychiatry day hospital, five from the oncohematology department, and nine from the treatment center for eating disorders). The interviews began with the photographs and followed an adaptation of the SHOWeD instrument [27]. The following questions were asked: What did you see here? What is happening? How does this represent your mental wellbeing? What do you think institutions (particularly educational and health institutions) could do to promote young people’s mental wellbeing? All interviews were conducted in person by a member of the research team in a quiet room at the hospital. During the interview, a healthcare professional familiar with the interviewee was present, who could intervene if the patient showed signs of discomfort but did not interfere with the content of the interview. The interviews had an average duration of 30 min (10 to 50 min), were digitally recorded, and transcribed verbatim.
d. Third, fourth, and fifth meetings. The third to fifth sessions of the workshop were dedicated to sharing photos of all participants (both those interviewed and the others) in groups to discuss them and find common themes related to mental wellbeing and suggestions for public institutions. The discussions were facilitated by two members of the research team and served to validate the content developed in the individual interviews. Each group then decided how to present the photos for the final exhibition, which was attended by representatives of institutions (schools and hospitals) and decision-makers in the field of youth health. After the exhibition, the photos were displayed in the respective departments of the hospitals at the request of the participants.

### 2.3. Data Analysis

The Photovoice workshop was conducted according to the structure outlined above, with all 39 participants in the form of group discussions based on the photos and collages they created. In order to ensure methodological consistency, only the photos submitted by the individually interviewed participants (*n* = 21) and the respective interview transcripts were used as data sources in this paper. Both the photos and the interview transcripts were subjected to an inductive thematic analysis using the Atlas.ti 24 software. Since the photos formed the starting point for the individual interview, they were inserted into the transcripts near the corresponding narratives and then analyzed together with the text.

Photo themes were developed by the research team in an iterative process. The group discussions that followed the individual interviews served to validate the photo themes with the participants. When analyzing the transcripts of the individual interviews, an inductive approach was followed in which the themes emerged from the data and were not guided by a pre-existing theoretical framework [28]. Theoretical models of psychological wellbeing were integrated in the subsequent interpretation phase. The following steps were followed: (1) Two researchers (F.T. and L.V.) independently read the transcripts, familiarized themselves with the data, and identified preliminary codes. (2) They met regularly with a third researcher (F.G.) to compare and redefine the codes. (3) They returned to the transcripts to apply the revised coding system and the percentage of agreement was 80%; discrepancies were resolved through discussions with the research team until a unanimous agreement was reached for all codes. (4) The codes were grouped into themes through discussion with the entire research team.

The final system of codes and themes is shown in Table 2 and Table 3. The quotations reproduced in this article were translated from Italian into English by a professional native translator, in constant discussions with the researchers to ensure that the content remained faithful to the original. Pseudonyms were used to preserve the anonymity of the participants.

## 3. Results

### 3.1. Mental Wellbeing

The thematic analysis was carried out on a total of 64 photos (realized by the 21 participants interviewed), which were assigned to the following 5 themes: nature (*n* = 24), relationships (*n* = 15), passions and leisure time (*n* = 9), animals (*n* = 8), and traveling (*n* = 8).

As for the analysis of the interview transcripts, a total of 259 quotes were coded under the following 8 themes identified to define mental wellbeing: sharing experiences and emotions with others (*n* = 62), calm and tranquility (*n* = 51), personal fulfilment (*n* = 32), sense of belonging (*n* = 30), pleasant physical sensations (*n* = 29), freedom and discovery (*n* = 29), involvement and commitment (*n* = 15), and happiness (*n* = 11). The results are presented below in relation to the photo themes (i.e., nature, relationships and animals, passion and leisure time, and traveling) and the mental wellbeing themes that were most strongly associated with each of them. The photos that best illustrate the suggested themes are included. Participants are identified by abbreviations indicating sex and age.

#### 3.1.1. Nature: Calm and Tranquility, Pleasant Physical Sensations, and Sense of Belonging

The photographs assigned to the theme of nature mainly showed landscapes, mountains, sea and beach views, the sky at dawn or dusk, and the night sky with the moon. These photographs were often associated with a definition of mental wellbeing, evoking the concepts of calm, tranquility, serenity, and peace. The images of the sky often convey this sense of peace, as can be seen in the following sentences:

“I like being outside at sunset, sitting and watching the sunset or the lights slowly coming on and the sun going down… it’s really a moment of a light heart, a heart at peace… of serenity”

(F, age 23)

“The moon has something to do with wellbeing, because looking at the moon is therapeutic. I take a break from my worries for a moment, put on my headphones and don’t hear any noise: I just look up at the sky and relax”

(M, age 17)

“I like watching the sky, it gives me a feeling of peace, of calm. Every sky is always different, the clouds change, every sky means a different feeling. That gives me calm”

(F, age 17)

**Figure 1:**
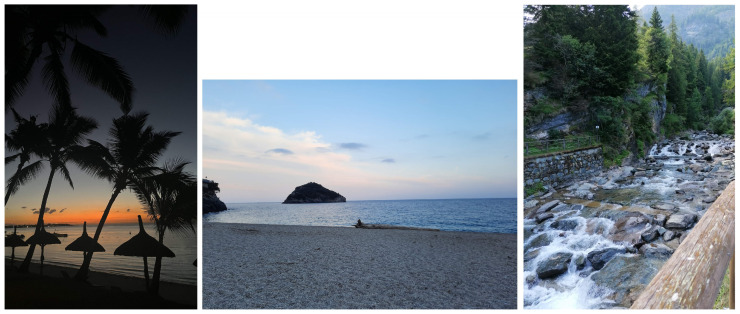
Examples of photos on nature and wellbeing: Mental wellbeing is watching a sunset (**left**). “Calm like the sea” (**middle**). Mental wellbeing is hearing the sound of water in the mountains (**right**).

Mountains or seascapes also evoke a feeling of wellbeing associated with tranquility. This was expressed by one participant who said:

“When I need a moment to switch off my mind, I go for a walk in the countryside or to the mountains or the sea, and in these places, I find a kind of… inner wellbeing, peace”

(F, age 25) (Figure 1)

A feeling of calm is very important for the mental wellbeing of young people, especially in today’s fast-paced society. Two participants said:

“I think if a person is not calm and quiet, but has a lot of thoughts, it is difficult for them to feel comfortable… so I think calmness is very important for young people’s health and wellbeing”

(F, age 15)

“I think it’s very important for mental wellbeing to spend some time… to relax… maybe observing nature, what’s around us, and not just being busy with your phone or social media”

(F, age 15)

Photographs depicting nature also evoked a definition of mental wellbeing associated with pleasant physical sensations; the following sentences are illustrative in this respect:

“[I photographed] this bright green expanse in the sunlight… it caught my eye because I stopped to observe and I really breathed in the air… there was the smell of grass and nature… it made me feel good”

(F, age 28)

“… just hearing the sound of the water… really gave me… a feeling of peace and wellbeing”

(F, age 20)

Another aspect of mental wellbeing that is particularly evoked by nature photos is a sense of belonging and connectedness to the outside world and an awareness of the present moment, especially in modern society characterized by frenetic rhythms. The following quotes support this theme:

“wellbeing for me means feeling my heart connected to what is around me, but at peace, i.e., at rest… wellbeing also means appreciating, enjoying and living in the here and now”

(F, age 23)

“[When I took this photo] I was looking for some kind of connection… with my feet, with the earth, with the ground, a moment of pause in which I can try to send away the negative thoughts, all the stress, suffering and pain of the day…”

(F, age 25)

For me, wellness is contact with nature… respecting the natural rhythms of people, not those of society …”

(F, age 28)

#### 3.1.2. Relationships and Animals: Sharing Experiences and Emotions

After the nature photos, the most numerous are those related to relationships, primarily with people, but also with animals. Both photo themes refer to the definition of wellbeing as sharing experiences and emotions with others. Examples of quotes that emphasize this theme are the following:

“All the photos I took at a time when I was in company, so someone was present who was important to me, so… the fact that maybe I can also associate wellbeing with the people around me…”

(F, age 17)

“This is the image of a table in a bar… it’s a pleasant moment of the day, spending a few hours with my friends at the bar, chatting and… it makes me feel good”

(F, age 19)

**Figure 2:**
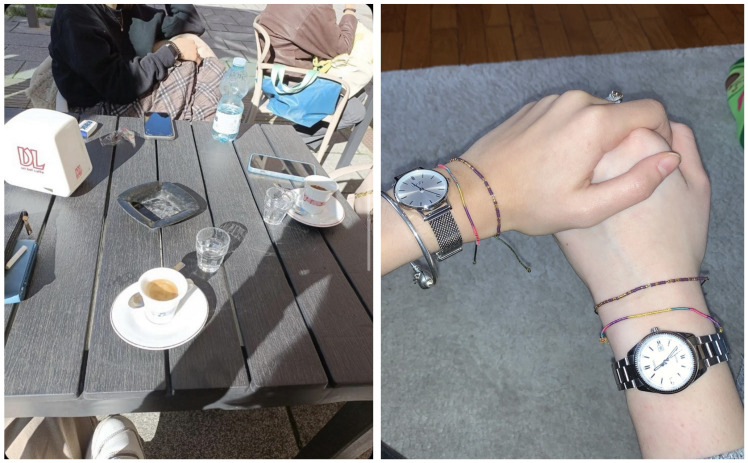
Examples of photos on relationships and wellbeing: Mental wellbeing is spending time at the bar with my friends (**left**). “Hand-in-hand” (**right**).

“Friends can be very helpful; they give us support when we feel fragile. Feeling good means feeling someone who gives comfort, I think of the picture hand in hand with my friend”

(F, age 17) (Figure 2)

**Figure 3:**
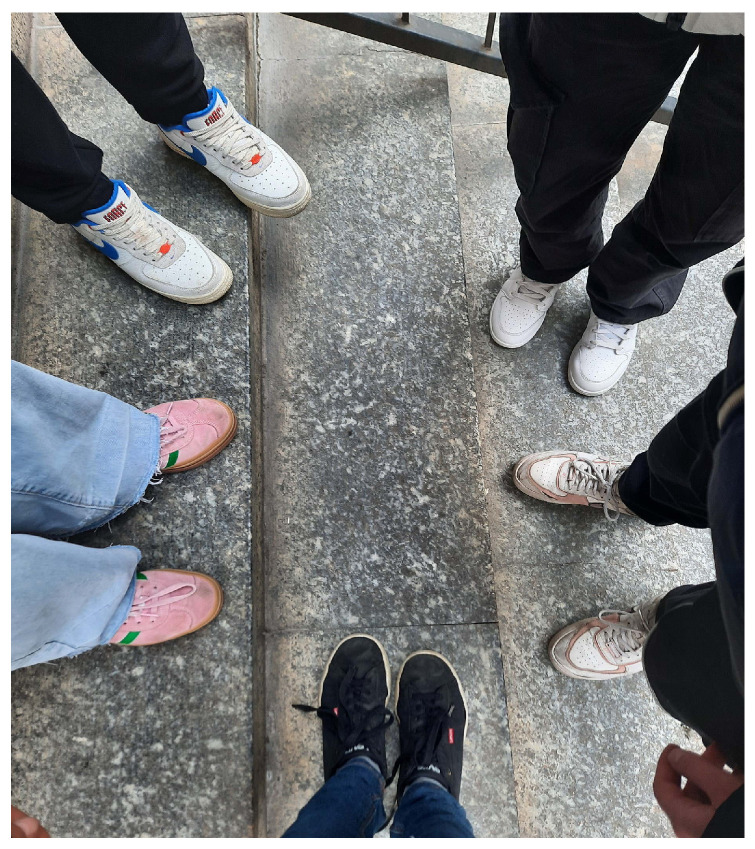
Examples of photos on relationships and wellbeing: “Chats between girlfriends”.

The presence of friends with whom to share experiences and emotions is an important source of wellbeing, especially in times of psychological fragility. One participant said:

“In this photo we were all together talking… it made me feel good… because I think friendships, especially real friendships which last for a long time, make you feel good… that is, a friend is a person you can tell anything to… you can let off steam, you can do whatever you want with your friend… even if you talk about negative things, you take these things away from yourself and then you feel better”

(F, age 15) (Figure 3)

Some participants redefined their relationships after the difficulties of the illness and maintained friendships with people who were close to them during times of suffering, considering these as real friendships. One participant said:

“Thanks to the illness, I realized what real friendships are that I will cherish all my life… these are the friends who are always there when you need them, even when you do not feel like talking, they call you, try to make your day… more than friends, I practically think of them as brothers”

(M, age 18)

**Figure 4:**
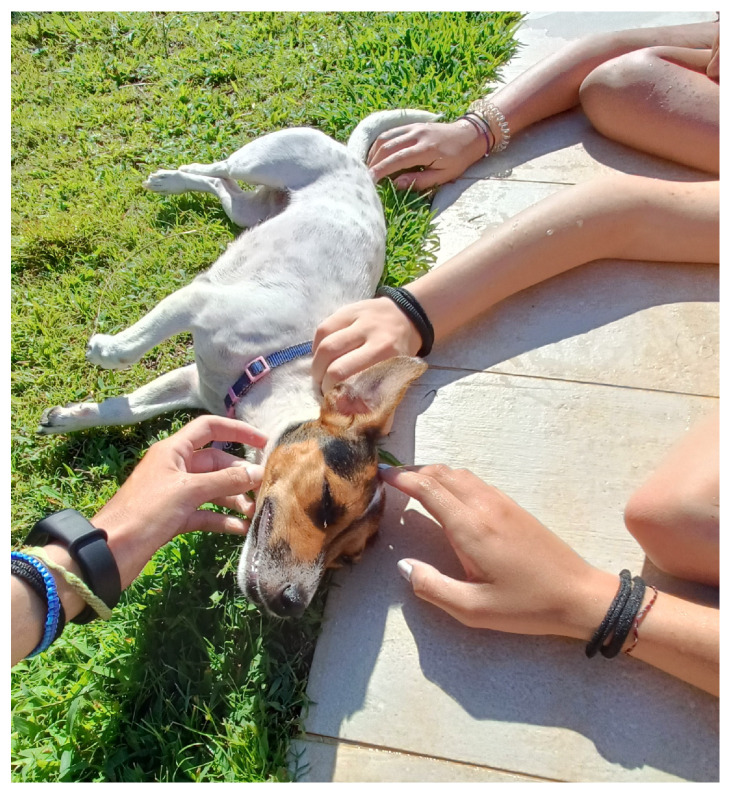
Examples of photos on animals and wellbeing: Mental wellbeing is having a pet.

The closeness and relationship with a pet is also a source of companionship and mental wellbeing, especially in difficult times, thanks to the comfort of physical contact (Figure 4). This is demonstrated by the following example quotes:

“Having an animal helps you feel better because they live their day in a simple and lively way” (F, age 17) “For example, when I was sick, I couldn’t go out, but my cat always slept next to me, she’s really present… even if it can’t replace a human, my cat makes me feel good every day”

(F, age 24)

#### 3.1.3. Passions and Leisure Time: Involvement, Commitment, and Personal Fulfilment

**Figure 5:**
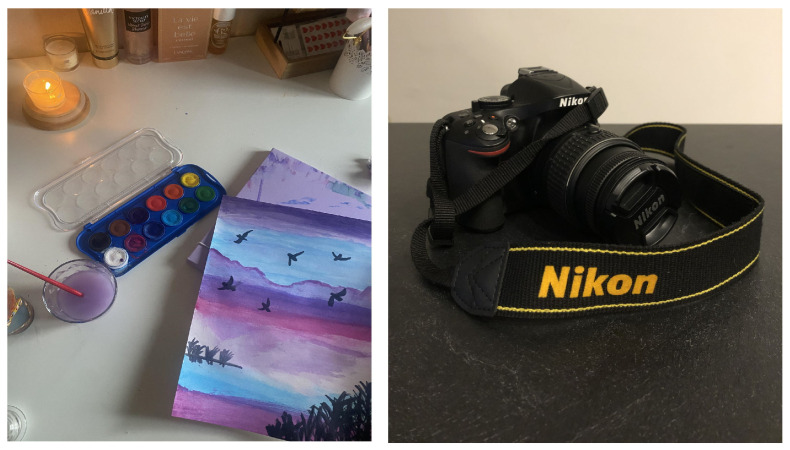
Examples of photos on passions and wellbeing: “Thoughts reset to zero” (**left**).Wellbeing is dedicating yourself to a photography course (**right**).

Photos related to passions represent activities such as reading, photography, painting, sports, and other leisure activities. They are related to a definition of wellbeing that consists primarily of involvement and engagement in pleasurable activities that provide an experience of total immersion, as well as caring for oneself (Figure 5). This was expressed by some participants as follows:

“Mental wellbeing is finding something in what we do that absorbs us”

(F, age 28)

“The photography course really gave me a feeling of wellbeing… to dedicate myself to an activity that I enjoy and that makes me feel good in the moment…”

(F, age 19)

“For me being well is also taking care of myself, for example going to the beautician, it makes me feel good”

(F, age 25)

Mental wellbeing is associated with a sense of personal fulfillment in which a person can experience self-satisfaction and self-confidence. The following sentences are examples:

“[wellbeing] means giving space to what we think and not to what others think, and giving ourselves more value, recognizing our own worth”

(F, age 20)

“[wellbeing] means being authentic, without fear of being alone or being judged by others, being able to feel good about yourself without necessarily needing someone else”

(F, age 22)

Furthermore, wellbeing has to do with emotions and, in particular, with the ability to deal with negative emotions. Two participants said:

“Mental health and wellbeing are strongly linked to emotions, which can sometimes be very numerous and simultaneous, and you also need to be able to control them”

(F, age 23)

“[wellbeing] is a state where we do not just have positive emotions, but a time where we embrace all emotions and manage them productively”

(F, age 20)

Finally, mental wellbeing is linked to a situation of balance, both between mind and body and between activities that one ‘has to do’ and those that one ‘chooses to do’. The following quotes support this theme:

“In my opinion… well being is… a bit of a balance between mind and body, and to achieve that, I think you have to work on yourself a bit”

(F, age 19)

“[wellbeing] is a balance between duty, for example going to school, and things that you enjoy, like I showed in the photo, so that you can also afford moments when you can be more relaxed and carefree”

(F, age 19)

#### 3.1.4. Traveling: Freedom, Discovery, and Happiness

**Figure 6:**
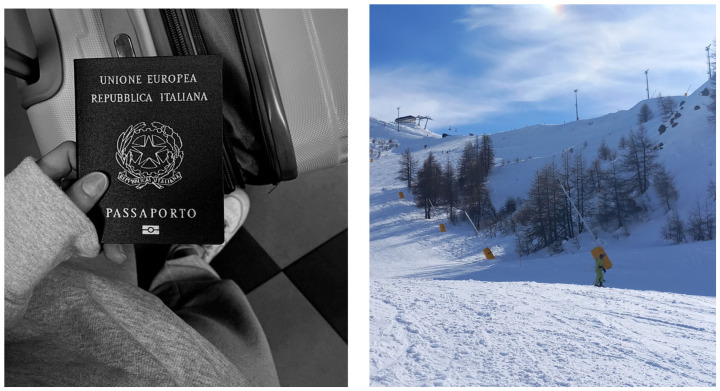
Examples of photos on traveling and wellbeing: Mental wellbeing is traveling (**left**). “I was happy to be back on skis” (**right**).

The last group of photos relates to the theme of travel, which was primarily associated with the definition of mental wellbeing as a sense of freedom. Freedom was defined as discovery and the search for novelty, as well as the absence of obstacles and external pressures. The following quotes emphasize this theme:

“This is a picture with a passport and a suitcase, because one of the things I like to do the most is traveling, discovering new things, going to new places and meeting people”

(F, age 19) (Figure 6)

“I really love traveling, it totally changes me, I mean… I feel happier, freer, I feel like a new person, it really gives me this feeling of wellbeing”

(F, age 19)

“Wellbeing is when you feel completely carefree, in the sense of free and just mentally lighter… without feeling burdened…”

(F, 21 years old)

“[wellbeing] is a person’s state in which they are free to make their own decisions, in the sense that they have no physical or mental limitations”

(F, age 19)

Finally, wellbeing is linked to the feeling of happiness, which is often experienced when traveling. However, happiness is also linked to the other photo themes mentioned above, in particular living out one’s passions and immersing oneself in nature. The value of these aspects is especially appreciated when one is able to return to their favorite activities after a period of illness or mental difficulty. The following quotes support this theme:

“Travel represents the idea of wellbeing because… when I travel, I feel better than when I am at home… Traveling always gives me a feeling of happiness, of wellbeing with myself and with others”

(F, age 19)

“It was practically my first real holiday with friends after my illness, my first sunrise by the sea, so I have a very happy memory of that morning…it was very nice”

(F, age 24)

“Here I wanted to immortalize my first day on skis after my illness, I liked this shot, there is the mountain, a slope, in a little forest… I was happy to be back on skis, it gives me a good” feeling”

(M, age 18)

### 3.2. Suggestions for Institutions to Promote the Mental Wellbeing of Young People

A total of 61 quotes from the interview transcripts were coded under the following six themes developed regarding proposals for institutions to promote mental wellbeing in young people: reducing levels of competition (*n* = 21), promoting inclusion (*n* = 15), promoting mental health awareness (*n* = 9), providing psychological support (*n* = 9), increasing outdoor activities (*n* = 5), and fostering collaboration between schools and health institutions (*n* = 2).

As outlined above, calmness and serenity are associated with mental wellbeing; reducing excessive competitiveness, performance anxiety, and stress is, therefore, seen as a fundamental measure to counteract psychological malaise and promote mental wellbeing. In order to improve wellbeing, participants believe that competition and pressure should be reduced both at school and at work, as the following quotes illustrate:

“Especially at school, the pressure is very high in my opinion…you only go to school and only think about grades and no longer about what you’re learning!”

(M, age 18)

“At school, but also at work, there is a lot of competition, a lack of mutual help, and in my opinion, this leads to performance anxiety, to great difficulty in asking for help, to fear of not being successful”

(F, age 19)

“The rhythm of work drives you to nervous exhaustion, to such a level of stress that you can no longer keep up with this rhythm… you feel incapable and may even become depressed”

(F, age 28)

To enhance mental wellbeing, institutions should recognize individual differences and promote inclusion of all people in schools, work environments, and society in general. The following quotes support this theme:

“Schools should value what each person can give and what each person is good at, because we are not all good at the same things”

(F, age 23)

“Institutions could do a lot to create a more positive, individualized working environment”

(F, age 21)

As far as school is concerned, many participants recognize that some teachers are sensitive and attentive to individual students and their difficulties, although much could still be accomplished for inclusion. This is illustrated by the following quotes:

“With some teachers it is possible to build a more human relationship, which I think is the best thing that helps both the teachers themselves and the students”

(F, age 19)

“School should be a safe place. Teachers should try to understand each individual student and not just look at the class as a whole”

(F, age 16)

“Many psychological aspects of a student are often pushed into the background in order to emphasize only academic performance”

(M, age 18)

As well as reducing competition and fostering inclusion, an important step in improving young people’s mental wellbeing should be to promote awareness of mental health. Proper information should be disseminated both in schools and in the wider social environment. These measures would be useful to combat the stigmatization of mental illness that is still prevalent in society. Two participants said:

“To increase wellbeing in any area, you should know what mental health is…. so definitely [give] information because a lot of people still do not know about it”

(F, age 19)

“Psychological support is often not socially accepted, although fortunately many young people no longer think that way”

(F, age 19)

Some participants have experienced these difficulties themselves, as the following quote shows:

“I experienced the ignorance of people who didn’t really know how to approach me and understand me because many didn’t know what illness or mental health meant… many people still can’t approach the subject in such a way that the ill person feels understood”

(F, age 19)

Another important measure to promote wellbeing would be the provision of psychological support in schools and health services. Two participants said:

“Psychological support at school should be increased and also offered at college”

(F, age 20)

“The possibility of having a reference psychologist, just as there is a pediatrician and a general practitioner for every citizen”

(F, age 25)

As mentioned above, nature can provide a sense of calm and serenity that promotes mental wellbeing. For this reason, some participants emphasized the importance of promoting group and outdoor activities in both schools and care settings.

“At school you should not spend all your time sitting at a desk, you should go out and explore nature. It gets you out of the house and helps you talk to new people. These are good experiences!”

(M, age 17)

“Give the green environment space. Because it is a place of peace and happiness for many”

(F, age 15)

“In hospital, you could do more activities outdoors and not indoors. When I was in hospital, we were locked in, we could not feel the air. We even wanted to have a small space. That was not possible for safety reasons, but we missed the outdoors”

(F, age 16)

“Group activities, creative activities, even outdoors… they are particularly useful for those who are in hospital for a long time to distract themselves”

(F, age 24)

Finally, some participants emphasized, albeit to a lesser extent, the importance of fostering collaboration between schools and health institutions. Two participants said:

“Schools should collaborate with health professionals and organize meetings, for example on sexuality or eating disorders”

(F, age 20)

“Teachers should train with health professionals and then pass this information on to their students”

(F, age 23)

## 4. Discussion

This paper reports the findings of a qualitative study based on Photovoice methodology conducted with a group of adolescents and young adults in a hospital context, which aimed to define the concept of mental wellbeing as perceived by participants and to explore with them what education and health institutions can do to promote adolescent wellbeing. We specifically included a sample of young people in a particularly fragile physical and mental state (i.e., patients with psychiatric and oncological pathology) to explore their specific view of mental wellbeing and their experiences of school and health services. Using the participatory action research approach, extensive photographic and text material was collected, discussed in detail with the participants, and analyzed through a thematic analysis. Based on the participatory definition of the concept of mental wellbeing, the Photovoice workshop enabled participants to give a “voice” to mental wellbeing measures that can be implemented by institutions and policy makers.

As for the definition of psychological wellbeing, the first prominent dimension was the need for calm and serenity. Academic and social pressure, excessive competitiveness, and anxiety were identified by the participants as the main causes of psychological discomfort, which is why they expressed a deep need for calm, peace, and serenity as sources of psychological wellbeing. This finding is consistent with evidence that children and adolescents reported increased levels of anxiety and depression following the COVID-19 pandemic [6,7,8]. Academic pressure is considered one of the main causes of increased mental health problems among youth [29]. It is interesting to note how this need for tranquility emerges above all from photographs of nature. The role of nature in adolescents’ psychological wellbeing is consistent with what has been shown in the literature for both adults [30] and young people [31]. Specifically, the natural environment promotes wellbeing through distraction from prolonged mental effort [32], recovery, restoration, stress reduction, and the promotion of positive emotions and self-regulation skills [33,34], as well as through increased self-esteem and social benefits [31].

A second salient aspect of psychological wellbeing is the relational dimension of sharing experiences and emotions with others. The role of positive relationships in psychological wellbeing is recognized in the main psychological multidimensional models of wellbeing, which are described in more detail in the following section, such as Ryff’s model [35,36] and Seligman’s PERMA model [37,38]. Humans are social beings by nature, and an important source of wellbeing is the experience of feeling valued, supported, and loved. The experience of satisfying and trusting relationships with others and the capacity for empathy and intimacy enrich an individual’s sense of worth and provide a source of social support that enhances wellbeing [35,36,37,38,39].

The relationship dimension is relevant to wellbeing at any age, but especially in adolescence and young adulthood, in relation to the main developmental task of this period of the life cycle, namely, the redefinition of identity, which occurs through negotiating independence from parents, a sense of belonging to the peer group, and engagement in romantic relationships [12]. As the participants noted, the relationship aspects are also crucial for wellbeing at school and university, the most important contexts of experience for adolescents and young adults. Wellbeing at school is not only linked to academic performance but also to the quality of relationships with peers and teachers. Schools become contexts of wellbeing when they are safe places that promote the inclusion of all students as well as school connectedness [12,40,41].

Interestingly, the need for safe relationships and affection arises not only from photos with people but also with animals, confirming the well-known findings on the role of animals in wellbeing [42,43,44], which forms the basis for animal-assisted therapy for hospitalized adolescents [45,46].

Apart from calm and relationships, the other dimensions of wellbeing indicated by the participants can be traced back to the two general perspectives of psychology on the definition of wellbeing, namely, the hedonic approach, which defines wellbeing in terms of the experience of pleasure and happiness, and the eudaimonic approach, which defines wellbeing by focusing on individual self-realization and a sense of personal fulfilment [11,47]. In our study, both dimensions emerged from participants’ words and photos: psychological wellbeing was indeed related to both hedonic aspects (i.e., pleasurable physical sensations and involvement in enjoyable activities, freedom, and happiness) and eudaimonic aspects (i.e., personal fulfillment and sense of belonging). The participants particularly recognized the importance of experiencing positive emotions and pleasant sensations as well as a feeling of freedom and independence from external constraints. On the other hand, they stated that it is important to achieve a sense of personal fulfillment through self-confidence, self-satisfaction, and the ability to manage negative emotions. This finding is consistent with studies that have emphasized the role of emotional self-efficacy for psychological wellbeing, particularly in early adolescent girls [48,49]. Other aspects of eudaemonic wellbeing included feeling immersed in meaningful activities, being aware of the present moment, and experiencing a sense of connectedness with the outside world.

All these aspects are reminiscent of some dimensions of Ryff’s [35,36] and Seligman’s [37,38] models of wellbeing. Specifically, Ryff’s model includes the following six dimensions: self-acceptance, personal growth, purpose in life, positive relations with others, environmental mastery, and autonomy. Seligman’s model, on the other hand, includes the following five dimensions: positive emotions, engagement, relationships, meaning, and accomplishments. In addition, some of the experiences of total immersion in pleasurable activities described by the participants reflect the concept of “flow” [50], i.e., an experience of optimal satisfaction in which the individual experiences a perfect combination of challenge and personal skills in the present moment. Our results on the linkages between voluntary and enjoyable activities, such as hobbies, arts, and sports, and psychological wellbeing add to the growing body of evidence of the mental health benefits of leisure activities [51]. Globally, the words used by some participants to describe psychological wellbeing seem to reflect the combined presence of hedonic and eudaimonic wellbeing components and the experience often referred to as “flourishing” [37,52].

Based on this complex definition of wellbeing, our participants provided useful suggestions for promoting mental wellbeing in adolescents and young adults. In response to the stress and academic pressures previously reported, participants’ suggestions seem to challenge public institutions, policy makers, and society to reflect on broader societal values that are closely linked to optimal performance and success. This paradigm, which is typical of many Western industrialized countries characterized by a competitive labor market and a high value placed on high expectations of success, can lead to an increased sense of malaise, particularly among people who experience physical or psychological difficulties. For this reason, there is increasing recognition of the need to address this issue as it impacts young people’s mental health [53].

Our participants call on institutions, especially schools, to pay attention to the individual characteristics of each student and the psychological aspects of students, especially those with the greatest difficulties. This would reduce pressures and create a truly inclusive environment that meets the definition of an inclusive education system that provides all students with an equal and participatory learning experience that meets their needs and preferences and enables them to develop their skills and realize their full potential [54].

Another important issue, according to our participants, is the lack of information about mental health. Although much has already been achieved in raising awareness of mental health in society, many steps still need to be taken, especially in the post-COVID-19 pandemic world. According to our participants, the stigmatization of mental illness needs to be tackled, and psychological support in schools, universities, and health settings needs to be improved. Nonetheless, healthcare professionals and policy makers should always be mindful of the impact that efforts to raise awareness of mental health can have at an individual and societal level. In particular, both the risk of over-pathologization and difficulties in accessing mental health services can exacerbate people’s distress and suffering [55]. International studies have called for these aspects to be taken into account and translated into effective policies adapted to the specificities of national contexts [56,57,58].

Another suggestion for the institutions is the expansion of outdoor activities in schools and the creation of open spaces in hospitals and health services. This suggestion echoes the need for nature, peace, and quiet that was repeatedly emphasized by participants to counteract the stress that leads to psychological discomfort. These suggestions are also consistent with evidence from the literature that emphasizes that interventions in contact with nature reduce stress, increase feelings of relaxation and tranquility, and promote wellbeing [59,60,61,62]. In particular, green spaces in hospitals (e.g., in pediatrics and oncology) can promote patient recovery and are beneficial for medical staff and patients’ relatives [63,64].

In summary, our data highlight participants’ awareness of the complexity of promoting wellbeing and the importance of collaboration between different institutions, which is not always easy to achieve.

### 4.1. Study Limitations

Our study has some limitations. The inclusion of a small sample of voluntary participants is typical of qualitative research and allows for a deeper exploration of the topic of interest. Of course, our findings must be considered in relation to the specific research context and can only be generalized to a limited extent, which is beyond the scope of qualitative participatory research. Future research should be replicated in other contexts to explore similarities and differences with our findings. In addition, our sample is predominantly female, leading to a potential gender bias, although this imbalance reflects epidemiologic data on female preponderance of mood, anxiety, and eating disorders [65]. Furthermore, potential bias may also be related to social desirability due to the institutional setting, although this was controlled by ensuring the conditions for participants’ freedom of expression while maintaining anonymity. The presence of the healthcare professional also served to make the interview as comfortable as possible. However, a possible social desirability effect cannot be ruled out, even if it was unintentional. Another limitation could be related to the heterogeneity of data collection. To ensure methodological coherence, the results of this study are based on a thematic analysis conducted using individual interviews with a subsample of workshop participants and their photographs. Data from group discussions based on photographs and collages were not recorded and, therefore, not analyzed thematically. This would have reduced the richness of our findings, although the themes of the group discussions largely overlapped with those of the individual interviews. Finally, as the concept of psychological wellbeing is culturally situated, it would be interesting to investigate this topic in different cultural contexts or by including people from different cultural backgrounds in group discussions.

### 4.2. Study Implications

Our study has methodological and practical implications. The novelty of the study lies in the use of participatory methodology with hospitalized adolescents. To the best of our knowledge, there is a lack of participatory studies on the mental wellbeing of adolescents and young adults who are vulnerable due to physical or psychiatric illness, especially in the post-pandemic period.

The Photovoice method was a useful tool for our study objectives, and despite the initial mistrust of some participants, the workshop was appreciated by all participants as a way to engage in issues that directly affect them and to make their voices heard by policy makers. Involving young patients as co-researchers is a resource-intensive process that requires intensive preparatory work and a high degree of flexibility [66]. It was precisely for the sake of this flexibility that we adapted our research to the four groups, e.g., we also recruited people outside the original age range who were interested in participating, we combined group discussions and audio recorded individual interviews, and we adapted the photo task, which is not allowed during hospitalization in child and adolescent psychiatry ward, by converting it into the creation of collages. Participants were involved as much as possible in all stages of the research process, including in the analysis of the photos and in the validation process of the data obtained from the interviews [67].

**Figure 7:**
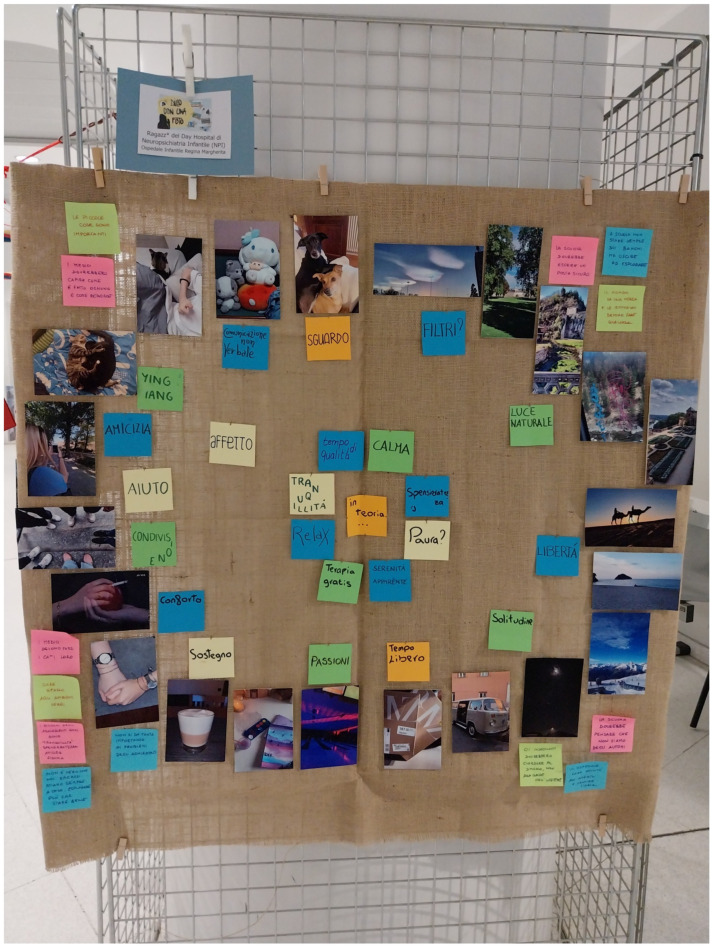
One of the posters displayed at the final exhibition.

The study is of practical importance in that it emphasizes that health policy should be based on the needs expressed by the target population. In terms of practical implications, the results of the study and the proposals for institutions generated by the participants were presented at a final meeting on the university campus, where the results of the wider participatory research were presented together with an exhibition of the materials produced (Figure 7). The conference was attended by representatives of local institutions and policy makers (from the fields of education, social policy, and health). Finally, the results of our study contributed to the development of recommendations for institutions, which were combined with evidence from the literature, relevant normative references, and suggestions for action. This study was, therefore, a starting point for the future development of strategies by health policy makers.

## 5. Conclusions

In this qualitative study, the Photovoice method was used to explore the construct of psychological wellbeing with a group of hospitalized adolescents and young adults and to develop suggestions for institutions to promote the psychological wellbeing of adolescents. According to our participants, psychological wellbeing is mainly associated with a state of calmness and serenity and satisfying social relationships. Mental wellbeing has both hedonic and eudaimonic aspects, which are related to happiness, pleasant sensations, and pleasure, as well as a sense of personal fulfillment, engagement, and a sense of purpose in life. Participants generated suggestions for public institutions and policy makers, as well as health professionals and adults concerned with the mental wellbeing of young people. Referring to their personal experiences, the participants emphasized that the following recommendations should be implemented by the institutions: (1) promoting inclusive educational and work contexts that respond to the specific characteristics of each adolescent and young adult and, in particular, reduce pressure to succeed, excessive competition, and performance anxiety; (2) promoting mental health awareness to combat the stigmatization of mental illness and promote psychological support services, especially in schools; (3) increasing outdoor activities in schools and health services, considering nature as the main source of stress relief and recovery; and (4) promoting collaboration between academic and health institutions.

## Figures and Tables

**Table 1:** Characteristics of study participants (*n* = 39).

	Sex	Age Range	Total *n*
Child and adolescent neuropsychiatry department	12 F, 1 M	13–16	13
Child and adolescent neuropsychiatry day hospital	10 F, 1 M	15–17	11
Oncohematology department	4 F, 2 M	18–24	6
Treatment center for eating disorders	9 F	19–28 *	9

* Note: Two 28-year-old patients were included even though their age was outside the range for inclusion because they were part of the day hospital patient group and were interested in participating in the workshop.

**Table 2:** Mental wellbeing: themes, codes, and sample quotations.

Themes	Codes	Sample Quotations
Sharing experiences and emotions with other people	Doing something you enjoy with friends	“Mental wellbeing means sharing the emotions of the present moment with another person”
	Experiences of mutual affection (with partners, family members, close friends, pets)	“Wellbeing is giving affection and feeling reciprocated”
	Empathy and comfort in difficult times	“Feeling good means feeling someone who gives comfort”
Calm and tranquility	Feeling of peace and serenity	“… the landscape with the snow around me really gave me serenity… I was really calm and happy… for me, that is mental wellbeing”
	Distraction from negative thoughts	“Mental wellbeing is to spend some time… to relax and banish negative thoughts”
Personal fulfilment	Self-satisfaction and self-confidence	“Mental wellbeing means recognizing your own value”
	Ability to manage negative emotions	“[Mental wellbeing means] having some control over your emotions and not being overwhelmed by negative emotions”
	Sense of balance	“Wellbeing is… a bit of a balance between mind and body”
Sense of belonging	Connection with the outside world (peace/calm can be named, but in explicit relation to the sense of connection with the outside world)	“For me, wellbeing means feeling connected to my surroundings, but at peace, i.e., at rest… wellbeing also means appreciating, enjoying and living in the here and now”
	Immersion in nature	“[Mental wellbeing] is being outdoors, in harmony with nature”
Pleasant physical sensations	Visual sensations	“[Wellbeing] is seeing a bright green meadow”
	Other sensations (tactile, auditory, olfactory)	“[Wellbeing] is feeling the contact with my cat”
Freedom and discovery	Absence of impediments and external pressure	“Wellbeing is when you feel completely carefree, free and simply mentally lighter… you don’t feel burdened”
	Search for novelty	“Discovering new things, going to new places… that makes me feel good…”
Involvement and commitment in enjoyable activities	Being absorbed in favorite activities	“In my opinion, skiing has something to do with wellbeing, because when you’re on skis, you concentrate on moving forward and have fewer thoughts, you concentrate on the sport and… and that gives you a good feeling”
	Taking care of oneself (without further specification indicating a sense of personal fulfilment)	“For me being well is also taking care of myself”
Happiness *	-	“[Mental wellbeing is] being happy about a small thing that may be worthless to others”

* Note: The code was not merged with other similar codes and is, therefore, considered a theme.

**Table 3:** Suggestions for institutions to promote youth wellbeing: themes, codes, and sample quotations.

Themes	Codes	Sample Quotations
Reducing the level of competition	Competition and anxiety at school	“In the school environment, perhaps… cause less anxiety, demand less performance from the students”
	Competition and anxiety in the workplace	“Competition should be reduced a little in the workplace”
Promoting inclusion	Inclusion at school	“The school should value what each individual can give and what each individual is good at”
	Inclusion in the workplace	“Institutions could do a lot to create a more positive, individualized work environment”
Promoting mental health awareness	Information on mental health at school	“Mental health awareness meetings should be organized in the school”
	Information on mental health in society	“To increase wellbeing in every area, people should be aware of what mental health is… so definitely information, because many people still don’t know what it is”
Providing psychological support	Psychological support at school	“Provide psychological support in schools and universities”
	Psychological support in health contexts	“Psychological support should be offered through the health service”
Increasing outdoor activities	Outdoor activities at school	“At school you shouldn’t sit in a classroom all the time, you should go out and do something…explore”
	Outdoor activities in health contexts	“In hospital, there should be more outdoor activities and not indoors”
Fostering collaboration between schools and health institutions *	-	“Schools should collaborate with health professionals”

* Note: The code was not merged with other similar codes and is, therefore, considered a theme.

## Data Availability

Data that support the findings of this study are available from the corresponding author upon reasonable request.
